# A Bead‐Based Screening Platform for Identifying Monoclonal Antibodies That Disrupt PD‐1/PD‐L1 Interactions

**DOI:** 10.1155/jimr/5189659

**Published:** 2026-03-31

**Authors:** Veridiane M. Pscheidt, Rodrigo B. Gassen, José E. Sacconi Nunes, Deise do Nascimento de Freitas, Valdir Barth, Fernanda B. Frozza, Milena D. Santos, Claudia P. Nunes, Cristina B. C. Bonorino

**Affiliations:** ^1^ Immunotherapy Laboratory, Federal University of Health Sciences of Porto Alegre, Porto Alegre, Rio Grande do Sul, Brazil, ufcspa.edu.br; ^2^ Department of Microbiology, University of São Paulo, São Paulo, São Paulo, Brazil, usp.br

**Keywords:** antibodies, flow cytometry, immune checkpoints inhibitors, immunotherapy, PD-L1

## Abstract

Monoclonal antibodies (mAbs) targeting immune checkpoint pathways such as programmed cell death protein 1 (PD‐1)/PD‐L1 are central to modern immunotherapy, yet scalable methods to assess their functional blockade remain limited. We present a bead‐based flow cytometry assay for quantifying the inhibition of PD‐1/PD‐L1 interaction by antibodies. Recombinant human PD‐1 protein was conjugated to polystyrene beads, and its interaction with recombinant human PD‐L1 protein labeled with a fluorochrome was measured. The inhibitory activity of an anti‐PD‐L1 mAb was quantified based on their ability to disrupt this interaction. The assay was validated for intra‐ and inter‐assay precision, in addition, functionality was confirmed using a T cell coculture assay. The assay demonstrated dose‐dependent inhibition by the αPD‐L1 mAb, with a calculated mean IC_50_ of 3.122 µg/mL. The method proved to be reproducible for the determination of antibody blocking activity, with relative standard deviation (RSD) < 20% between three independent runs. At the concentration approximating the IC_50_ detected on the bead assay, the antibody significantly restored CD69 expression on the T cell surface (*p* = 0.0001) in a coculture in vitro system. In addition, the methodology could successfully distinguish the blocking capacity of two anti‐PD‐L1 antibodies with different affinities. This high‐throughput compatible platform offers a reliable tool for screening PD‐1/PD‐L1 blocking antibodies, supporting immunotherapy discovery and development.

## 1. Introduction

Cancer remains the second leading cause of death globally, accounting for ~10 million deaths in 2020—nearly one in six worldwide [1]. Immunotherapy is revolutionizing cancer treatment, particularly through the clinical success of immune checkpoint inhibitors (ICIs) targeting negative regulatory pathways in T cell activation [2]. Among the most prominent targets are the programmed cell death protein 1 (PD‐1) and its ligand PD‐L1, a pathway that plays a central role in tumor immune evasion [3]. PD‐1 is primarily expressed on activated T cells, and upon engagement with PD‐L1 on tumor cells or antigen‐presenting cells (APCs), T cell function is attenuated [4, 5]. Blocking this interaction using monoclonal antibodies (mAbs) can restore T cell function and enhance antitumor immunity [6]. Due to their specificity and stability [7], mAbs have become a cornerstone of immunotherapeutic development, driving rapid expansion of the global antibody therapeutics market, which reached USD 237.64 billion in 2023 with an expected annual growth rate of 11% [8]. Given the rising demand, there is a growing need to develop new antibody sequences and biosimilars to ensure therapeutic availability, reduce production costs, and expand global access to immunotherapy. A critical component of this pipeline is the functional characterization of candidate mAbs for their ability to inhibit immune checkpoint interactions. Flow cytometry is a widely accessible, rapid, and sensitive technique used in both research and clinical settings. However, relatively few flow cytometry‐based methods are available for the quantitative assessment of immune checkpoint blockade. Here we have described a flow cytometry assay to assess blocking activity of antibodies targeting immune checkpoint proteins. Using fluorescently labeled PD‐1 and PD‐L1 proteins, we measure the capacity of an anti‐PD‐L1 mAb to block PD‐1/PD‐L1 binding, providing a tool to estimate their therapeutic potential in the development of new mAbs. This assay demonstrated dose‐dependent inhibition by a reference αPD‐L1 mAb, showed reproducibility across independent experiments, and correlated with functional restoration of T cell activation in a coculture assay. Together, these findings establish a robust and scalable method for evaluating the inhibitory potential of PD‐1/PD‐L1 blocking antibodies, offering a valuable tool for immunotherapy development and screening.

## 2. Methods and Materials

### 2.1. PD‐1 Bead Conjugation and Confirmation

The recombinant human PD‐1 protein (Cat. #Z03424, GenScript) was conjugated to functional beads using the BD Cytometric Bead Array (CBA) Functional Bead Conjugation Buffer Set (Cat. #558556, BD), following the manufacturer’s instructions. Briefly, 75 µL of functional beads A4 (Cat. #558578, BD) were vortexed, sonicated, and incubated with 1 M dithiothreitol (Cat. #20290, Thermo Fisher Scientific) for 1 h at room temperature on an orbital shaker, protected from light. The beads were washed three times with the coupling buffer and resuspended in 20 µL of the same buffer. In parallel, 22.5 , 45, and 90 µL of PD‐1 protein suspended in phosphate‐buffered saline (PBS) 1 × (Cat. #590338, Laborclin) at 1 mg/mL were modified by adding 0.5 µL, 1 µL, and 2 µL, respectively, of sulfo‐SMCC (Cat. #M6035–10 MG, Thermo Fisher Scientific) at 2 mg/mL, followed by 1 h incubation on an orbital shaker at room temperature. Unreacted components were removed by buffer exchange using a spin column (Cat. #28918004, Global Life Sciences) equilibrated with coupling buffer, and the eluted protein was immediately added to the prepared bead suspension. After vortexing, the mixture was incubated for 1 h in an orbital shaker at room temperature, followed by the addition of 2 µL of N‐ethylmaleimide (Cat. #23030, Thermo Fisher Scientific) at 2 mg/mL, and 15 min incubation at the same conditions. The beads were pelleted, and the supernatant collected to determine indirect PD‐1 bead‐conjugated concentration. Next, beads are washed three times with the storage buffer, centrifuged at 900 × *g* for 3 min, and resuspended in 0.5 mL of storage buffer, yielding a final concentration of 6 × 10^6^ beads/mL. The conjugated beads were stored at 4°C and protected from light. Conjugation was confirmed by incubating the beads with BV421 anti‐PD‐1 antibody clone MIH4 (Cat. #564323, BD) followed by flow cytometry analysis in a BD FACSCanto II flow cytometer. The data were analyzed in the FlowJo 10.7.1 software. Successful conjugation was indicated by a signal exceeding 500 MFI compared to the negative control (beads not conjugated with protein).

### 2.2. Quantification of PD‐1 Protein Conjugated With Beads

The concentration of PD‐1 protein conjugated to the beads was determined by an indirect way, measuring the concentration of PD‐1 unconjugated excess in the supernatant collected as described above. Briefly, the supernatant postconjugation reaction was collected, and a buffer exchange using a spin column was performed (Cat. #28918004, Global Life Sciences). The eluate was used to quantify PD‐1 protein using the Qubit Protein Assay (Cat. #Q33211, Thermo Fisher Scientific) following the manufacturer’s instructions. Briefly, a working solution was prepared by diluting the Qubit protein reagent 1:200 in Qubit protein buffer. 2 –5 µL of sample were added to 198 –195 µL of the working solution in Qubit assay tubes (Cat. #Q32856), vortexed gently for 2–3 s, and incubated at room temperature for 15 min protected from light. Fluorescence was measured using the Qubit 4 Fluorometer, and protein concentrations were calculated based on a standard curve, generated with the kit bovine serum albumin (BSA) standards.

### 2.3. PD‐L1 Protein Labeling With Fluorescence

Alexa Fluor 488 (AF 488) conjugation with human PD‐L1 protein (Cat. #10084‐H08H, Sino Biological) was performed using a commercial Alexa Fluor 488 carboxylic acid tetrafluorophenyl ester kit (Cat. #A10235, Invitrogen). Briefly, PD‐L1 protein (1 mg/mL) was stirred with 1 M sodium bicarbonate in the vial of reactive dye for 1 h at room temperature. The reaction mixture was purified according to the specifications of the manufacturer. Following this, the protein was aliquoted and stored at 20°C. Quantification of protein labeling was performed with the NanoDrop Lite spectrophotometer (Thermo Fisher, USA).

### 2.4. PD‐L1 AF488 Titration With PD‐1 Functionalized Beads

Beads conjugated with PD‐1 were diluted 50× in wash buffer (PBS 1×, 0.5% fetal bovine serum (FBS), and 0.05% polysorbate 20). Approximately 6000 beads of this dilution were added to each well of a 96‐well plate. The PD‐L1 AF488 protein was serially diluted in the stain buffer (Cat. #554656, BD Biosciences) to concentrations of 12.5–3.12 µg/mL per well and then added to diluted beads, mixing up and down. The plate was incubated for 1 h at 37°C. After incubation, the plate was washed three times with the wash buffer and centrifuged at 1500 rpm for 5 min. The supernatant was discarded by inversion after each centrifugation. The beads were resuspended in 150 µL of the wash buffer and transferred to a flow cytometer tube. Approximately 5000 events were acquired in a BD FACSCanto II flow cytometer. The PD‐L1 binding to PD‐1 covered bead data were analyzed in the FlowJo 10.7.1 software. The assay specificity was evaluated using a competitive binding assay. Additionally, to evaluate the specificity of the interaction, PD‐1‐conjugated beads were preincubated with unlabeled PD‐L1 (25 µg/mL) for 30 min at room temperature. The beads were then washed and incubated with 12.5 µg/mL of PD‐L1 AF488 for 1 h at 37°C. To evaluate the stability of the interaction PD‐1/PD‐L1 AF488 over the time, following the staining protocol, the samples were stored at 4°C protected from light, and the percentage of binding and the MFI were analyzed at different time points: immediately, 3, 20, and 24 h poststaining.

### 2.5. Flow Cytometry Blocking Assay

The approach to detect blocking activity of antibodies against PD‐1/PD‐L1 immune checkpoints using flow cytometry consists of measuring the interaction between the PD‐L1 protein conjugated with fluorescence (AF488) and the PD‐1 receptor conjugated in polystyrene beads. When the PD‐L1 AF488 is preincubated with a blocking α‐PD‐L1 mAb, the fluorescence signal is reduced or absent in comparison to that observed with a nonblocking antibody (isotype control). Specifically, beads conjugated with PD‐1 were diluted 50× in the wash buffer (PBS 1×, 0.5% FBS, and 0.05% polysorbate 20). Approximately 6000 beads of this dilution were added to each well of a 96‐well plate, followed by the addition of 150 µL of wash buffer. The plate was centrifuged at 1500 rpm for 5 min at 4°C, and the supernatant was discarded by inverting the plate. In another 96‐well plate, the PD‐L1 AF488 protein was diluted to a final concentration of 12.5 µg/mL in the stain buffer (Cat. #554656, BD Biosciences, USA) per well. Anti‐human PD‐L1 (αPD‐L1) mAb biosimilar to atezolizumab (Cat. #hpdl1‐mab1, InvivoGen, USA), avelumab (Cat. #SIM0021, Bioxcell), αPD‐L1 mAb nonblocking (Cat. #10084‐R001, Sino Biological), or a polyclonal human IgG control (Cat. #I4506, Sigma, USA) were diluted in a stain buffer at a final concentration of 0.1, 1, 5, and 10 µg/mL per well and mixed with the PD‐L1 AF488 in the plate. The plate was incubated for 30 min at room temperature. The antibody–protein PD‐L1 AF488 mix was then added to the PD‐1 beads, mixing up and down. The plate was incubated for 1 h at 37°C. After incubation, the plate was washed three times with the wash buffer and centrifuged at 1500 rpm for 5 min, and the supernatant was discarded by inversion after each centrifugation. The beads were resuspended in 150 µL of wash buffer and transferred to a flow cytometer tube. Approximately 5000 events were acquired in a BD FACSCanto II flow cytometer. The data were analyzed in the FlowJo 10.7.1 software.

### 2.6. Construction and Cloning of Human PD‐L1 Plasmid

Total RNA was extracted from Expi293F cells (Cat. #A14527, Invitrogen, USA) using TRI reagent (Cat. #T9424, Sigma–Aldrich). The cDNA was constructed using the GoScript Reverse Transcription System with random primers (Cat. #A5000, Promega) followed by PCR amplification using Q5 High‐Fidelity DNA Polymerase (Cat. #M0491, New England Biolabs) and gene‐specific primers, forward sequence 5′ GGCATTCCAGAAAGATGAGG 3′ and reverse sequence 5′ CCCTGCTTGAAGATCAGAAGTTCC 3′, synthesized by Exxtend, Brazil, according to manufacturers’ protocols. This PCR product was used as a template in a nested PCR reaction using oligos 5′acgactcactataggctagcATGAGGATATTTGCTGTCTTTATATTC 3′ and 5′ ccgcccgggtcgactctagaTTACGTCTCCTCCAAATGTG 3′ synthetized by Exxtend, Brazil, in order to add a complementary sequence to the plasmid. The PD‐L1 DNA amplification was confirmed with an agarose gel electrophoresis; the band with ~907 pb was excised and recovered using a commercial kit (Cat. #D4001, Zymo Research). The PD‐L1 insert was assembled into the pCI‐neo plasmid (#E1841, Promega) with NEBuilder Hifi DNA Assembly (# Cat. E2621X, New England Biolabs). The assembled plasmid was transformed into *Escherichia coli* DH5α competent cells via heat shock. Three colonies were selected and grown in LB medium. Plasmid DNA was extracted and purified using a miniprep kit (Cat. #D4209, Zymo Research). Sanger sequencing was performed to confirm insert identity, and the clone with the highest sequence identity to the reference *CD274* gene codifying the PD‐L1 protein was selected for further experiments. The human PD‐L1 plasmid (pCI‐neo‐hPD‐L1) and the empty plasmid (pCI‐neo) were cloned and expanded in *E. coli* DH5*α* through thermal shock transformation. The plasmid DNA was purified using a midiprep kit (Cat. #D4201, Zymo Research) and quantified using a NanoDrop Lite spectrophotometer; the DNA was stored at ‐20°C.

### 2.7. Expression of Human PD‐L1 in Expi293F Cells Surface

Expi293F cells cultured in expression medium (Cat. #A1435101, Thermo Fisher Scientific) were transfected with pCI‐neo‐hPD‐L1 DNA using Lipofectamine 3000 (Cat. #L3000008, Thermo Fisher Scientific) at a 1:1 ratio of plasmid DNA to lipofectamine, with Opti‐MEM Reduced Serum Medium (Cat. #31985062, Thermo Fisher Scientific), according to the manufacturer’s instructions. Cells transfected with the pCI‐neo empty vector were used as the negative control. On the following day, transfected cells were selected for resistance to the G418 antibiotic (Cat. # 11811031, Gibco), conferred by the pCI‐neo vector, using a final concentration of 850 μg/mL. Selection was maintained for 23 days. PD‐L1 surface expression was confirmed by flow cytometry. Briefly, ~1 × 10^6^ Expi293F cells transfected with either pCI‐neo‐hPD‐L1 or the empty pCI‐neo vector were pelleted by centrifugation at 1500 rpm for 5 min, and the supernatant was discarded. Then, the cells were stained with the BD Horizon Fixable Viability Stain 510 (Cat. #564406, BD) diluted 1:1000 in PBS 1 × for 15 min at room temperature. After, the cells were washed with PBS 1 × and pelleted by centrifugation at 1500 rpm for 5 min, followed by incubation with human Fc block (clone Fc1) (Cat. #564219, BD) diluted 1 :1000 in stain buffer (Cat. #554657, BD) for 20 min at room temperature. Cells were then stained with PE‐Cy7 mouse anti‐human CD274 (clone MIH1) (Cat. #558017, BD) or isotype control PE‐Cy7 (clone MOPC‐21) (Cat. #557872, BD) for 30 min at room temperature. After, the cells were washed twice with the stain buffer, centrifuged at 1500 rpm for 5 min and resuspended in the stain buffer. Flow cytometry analysis was performed using a BD FACSCanto II cytometer and FlowJo 10.7.1 software.

### 2.8. Peripheral Blood Mononuclear Cells (PBMCs) Isolation and Sorting of CD3^+^ Cells

This study was approved by the Research Ethics Committee of Irmandade Santa Casa de Misericórdia de Porto Alegre (ISCMPA), under Protocol Number 52266121.0.0000.5335. After written informed consent, 20 mL of peripheral blood was collected from a healthy adult donor over and placed in EDTA tubes. PBMCs were isolated by density‐gradient centrifugation at 1500 rpm for 20 min using Ficoll‐Paque PLUS (Cat. #17144002, Cytiva). The PBMC layer was collected and washed with PBS 1×. Next, PBMCs were stained with the BD Horizon Fixable Viability Stain 510 (Cat. #564406, BD) diluted 1 : 1000 in PBS 1× for 15 min at room temperature. Cells were washed with stain buffer (Cat. #554657, BD) and then stained with APC‐H7 mouse anti‐human CD3 (clone SK7) (Cat. #641415, BD) for 30 min at room temperature. PBMCs were washed twice with stain buffer, centrifuged at 1500 rpm for 5 min and resuspended in 8 mL. A total of 50,000 viable CD3^+^ cells were sorted per well into a 96‐well plate using the BD FACSMelody cell sorter (purity mode). Sorting purity was assessed using FlowJo 10.7.1 software, consistently yielding populations with over 98% purity (Figure S2).

### 2.9. Coculture Assay

Expi PD‐L1^+^ cells were cocultured with human CD3^+^ T lymphocytes at a 2:1 ratio. Briefly, 1 × 10^5^ Expi PD‐L1^+^ cells were diluted in RPMI‐1640 medium (Cat. #R6504, Sigma–Aldrich) supplemented with 10% FBS (Cat. #12657029, Thermo Fisher Scientific), and αPD‐L1 (Cat. #hpdl1‐mab1, InvivoGen) or isotype control IgG_1_ (Cat. #I4506, Sigma) at a final concentration of 6 μg/mL was added. This suspension was added to 5 × 10^4^ CD3^+^ cells previously plated in RPMI‐1640 + 10% FBS. Next, 1 μL of anti‐CD3 (clone UCHT1) (Cat. #555329) and 1 μL of anti‐CD28 (clone CD28.2) (Cat. #555725, BD) were added to each well. The final coculture volume was 200 μL per well, with a final antibody concentration of 3 μg/mL for αPD‐L1 or isotype control. Cells were incubated at 37°C with 5% CO_2_ for 18 h.

### 2.10. Immunophenotyping and Intracellular Staining

Following 18 h of coculture with Expi PD‐L1^+^ cells and human CD3^+^ T lymphocytes, cells were collected by centrifugation at 1,500 rpm for 5 min. The supernatant was discarded and cells were washed with PBS 1×. Next, they were stained with the BD Horizon Fixable Viability Stain 510 (Cat. #564406, BD) diluted 1:1000 in PBS 1× for 15 min on ice. Then, cells were washed and resuspended with Fc block (clone Fc1) (Cat. #564219, BD) diluted 1:1000 in the stain buffer, followed by incubation for 20 min on ice. Surface staining was performed by adding 50 μL of an antibody cocktail composed of anti‐human CD45 (clone HI30, Cat. #555484, BD), CD3 (clone SK7, Cat. #641415, BD), CD4 (clone SK3, Cat. #557852, BD), CD8 (clone HIT8a, Cat. #555634, BD), CD69 (clone FN50, Cat. #555533, BD), and PD‐1 (clone MIH4, Cat. #564323, BD). Cells were incubated for 30 min on ice and in the dark. After, the cells were washed with the stain buffer, and the fixation/permeabilization solution of an intracellular staining kit was added (Cat. #00‐5523‐00, Invitrogen), following the manufacturer’s instructions. Cells were incubated for 30 min on ice and in the dark. Then, a 1 × permeabilization buffer was added, and cells were centrifuged again. Next, 100 μL of intracellular antibody cocktail diluted in 1 × permeabilization buffer with anti‐human IFN‐γ (clone 4S.B3, Cat. #563416, BD) and Ki‐67 (clone B56, Cat. #567719, BD) was added per well. Cells were incubated for 40 min at room temperature in the dark. Finally, cells were washed, and a total of 30,000 events per sample were acquired on a BD FACSymphony A5 flow cytometer. Data analysis was performed using FlowJo 10.7.1 software. Antibody dilutions are available in Table S1.

### 2.11. Determination of Intra‐ and Inter‐Assay Precision

To assess inter‐assay precision, the dose–response curve of the αPD‐L1 mAb was analyzed in three independent experiments performed on different days and with two different analysts. Intra‐assay precision was determined from the results of six replicates per concentration point ranging from 0.1 to 15 μg/mL in a single run. Precision was measured as the percent relative standard deviation (%RSD) for each concentration point. The data were analyzed in two ways, as a normalized binding signal (geometric mean fluorescence intensity [gMFI]/gMFI_max_) and as an inhibition rate (1 − gMFI/gMFI_max_). The half‐maximal inhibitory concentration (IC_50_) was calculated for each independent run.

### 2.12. Statistical Analysis

Comparisons between two experimental groups were analyzed using an unpaired *t*‐test, while comparisons among three or more groups were performed using a one‐way ordinary ANOVA followed by Tukey’s multiple comparisons test. Nonlinear regression analysis was used to model both protein binding and inhibition data. Saturation binding curves were analyzed to characterize the dose‐dependent interaction between PD‐1 and PD‐L1 AF488, while dose–response inhibition curves were fitted using a four‐parameter variable slope model to calculate the half‐maximal inhibitory concentration (IC_50_) values. A *p*‐value of less than 0.05 was considered statistically significant for all tests. All statistical analyses and graph generation were performed using GraphPad Prism software (Version 9.0.2, GraphPad Software, Inc.).

## 3. Results

### 3.1. Development and Characterization of a PD‐1 Functionalized Bead‐Based Assay

To establish a quantitative assay for PD‐1/PD‐L1 interaction, we covalently coupled recombinant human PD‐1 protein to polystyrene microspheres (Figure 1A). Varying input amounts of PD‐1 (22.5, 45, and 90 µg) were evaluated to optimize conjugation efficiency. Indirect quantification of immobilized PD‐1—calculated by subtracting the unbound protein in the supernatant from the initial input—revealed a linear correlation between protein input and conjugation yield. Notably, the 90 µg input condition yielded the highest coupling efficiency, with > 50% of total PD‐1 retained on the bead surface (Figure 1A). Flow cytometric validation of surface expression using an anti‐PD‐1 antibody confirmed conjugation, with a gMFI of 99,480 (Figure 1B), being selected for all subsequent assays.

Figure 1Construction and characterization of PD‐1‐coated beads for evaluating PD‐L1 binding. (A) Schematic of a polystyrene bead coated with recombinant human PD‐1 and indirect quantification of PD‐1 conjugation on the bead surface, measured by a fluorometer. (B) Flow cytometry analysis of beads conjugated with different amounts of recombinant human PD‐1 protein (22.5, 45, and 90 µg) reported in percentage of positive beads and geometric mean intensity fluorescence (gMFI). (C) Schematic illustrating the binding of soluble PD‐L1AF488 to the PD‐1‐coated bead. (D, E) Nonlinear regression dose–response analysis of PD‐L1 AF488 binding to PD‐1‐coated beads. (D) Binding curve expressed as the ratio of geometric mean fluorescence intensity (gMFI) to the maximum gMFI (gMFI/gMFI_max_) and (E) percentage of binding. (F) Assessment of binding specificity using unconjugated beads, incubated with the highest tested concentration of PD‐L1 AF488 (12.5 μg/mL). (G) Validation of binding specificity. The specificity of the PD‐1/PD‐L1 interaction was confirmed by a self‐competition assay (PD‐L1 + PD‐L1 AF488). (H, I) Stability of the PD‐L1 AF488 and PD‐1 beads at time points 0, 3, 20, and 24 h after staining. (H) The graph displays the normalized fluorescence intensity (MFI/MFI_max_) alongside (I) the percentage of positive beads over time. Data represent mean ± SD. Representative data from three independent experiments with *n* = 3.

**Figure:**
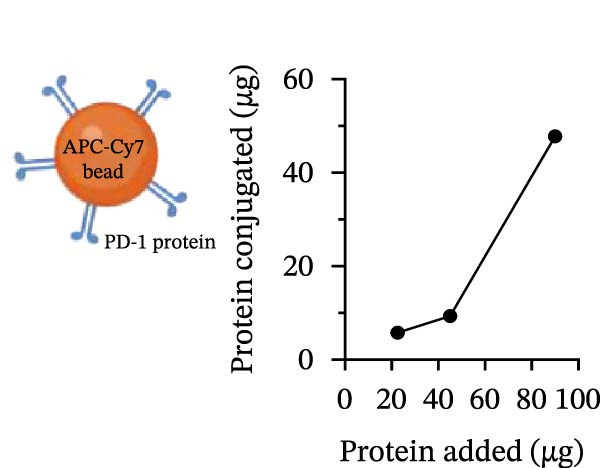
(A)

**Figure:**
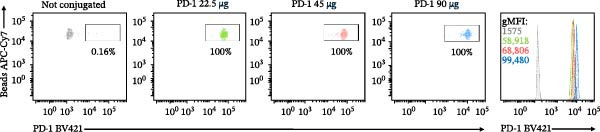
(B)

**Figure:**
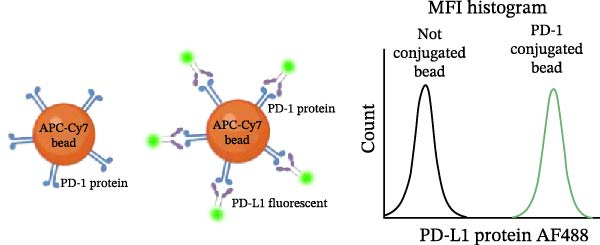
(C)

**Figure:**
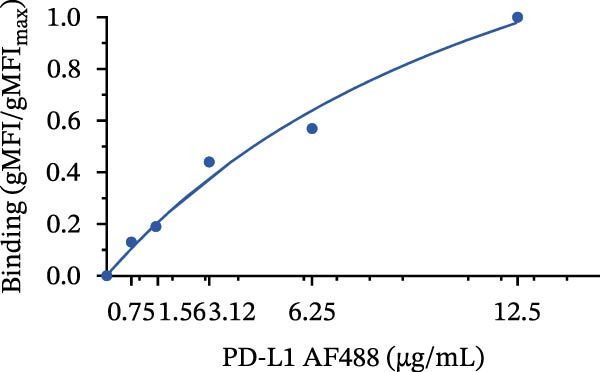
(D)

**Figure:**
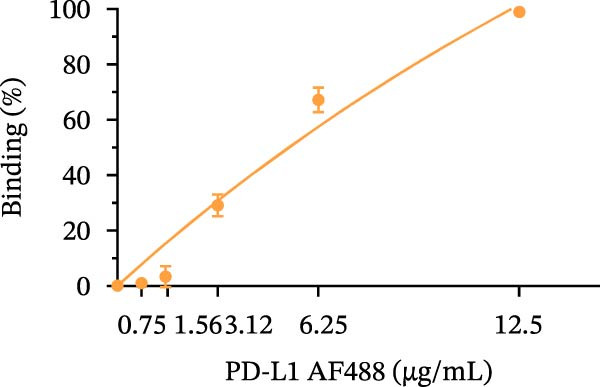
(E)

**Figure:**
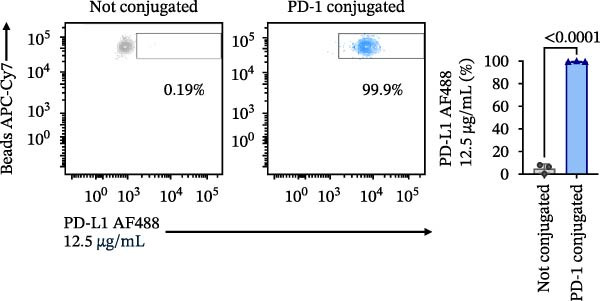
(F)

**Figure:**
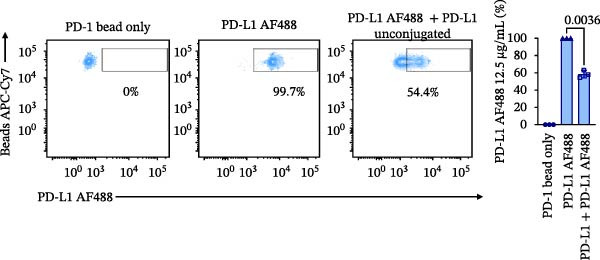
(G)

**Figure:**
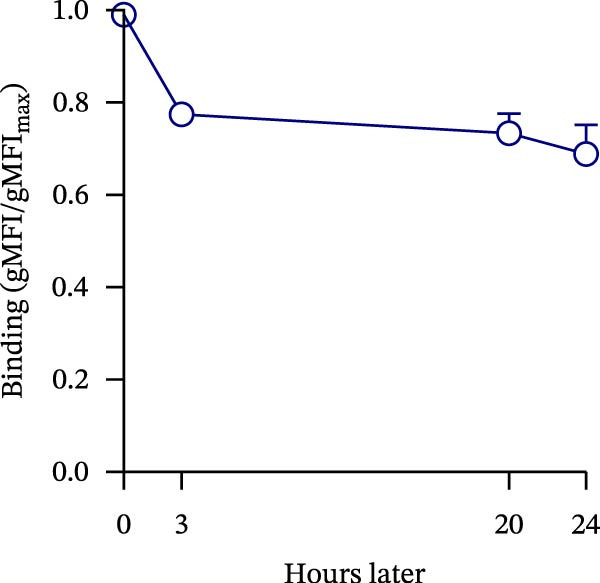
(H)

**Figure:**
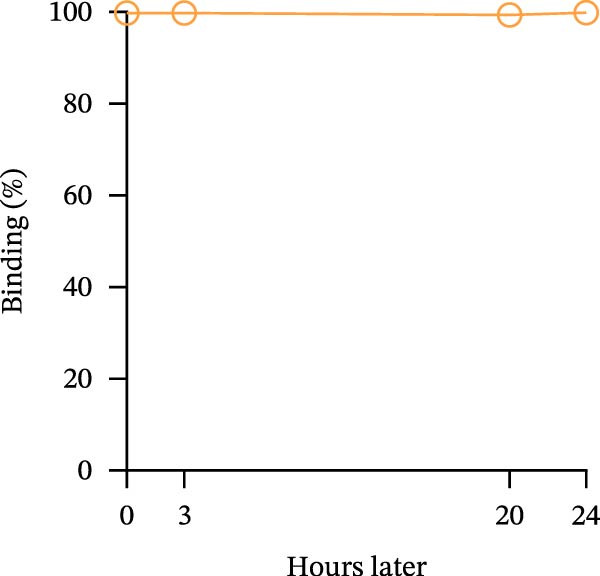
(I)

Once the PD‐1‐coated bead platform was finished, we evaluated its ability to specifically bind the PD‐L1 protein labeled with the AF488 fluorophore (PD‐L1 AF488), as schematized in Figure 1C. The PD‐1 beads were incubated with increasing concentrations of PD‐L1 AF488 : 0.75, 1.56, 3.12, 6.25, and 12.5 µg/mL. Nonlinear regression analysis revealed dose‐dependent and saturable binding both in gMFI (Figure 1D) and percentage of binding (Figure 1E). Finally, to ensure the specificity of the observed interaction, we evaluated the binding using unconjugated beads as the negative control and the highest tested concentration of PD‐L1 AF488 (12.5 μg/mL). As demonstrated in Figure 1F, PD‐1‐coated beads exhibited significant PD‐L1 binding when compared to the unconjugated beads (*p*  < 0.0001). To confirm the specificity of this interaction, we performed a self‐competition assay (Figure 1G), where preincubation with unlabeled PD‐L1 inhibited significantly the fluorescent signal by around 40% (*p* = 0.036). To evaluate the platform robustness for high‐throughput screening, we assessed the temporal stability of the signal at 4°C. We monitored both the MFI and the frequency of positive beads. While a reduction around 20% in MFI at first 3 h and 30% over 24 h was observed (Figure 1H), the percentage of positive beads remained unchanged (>98%) across all time points (Figure 1I). This demonstrates that despite minor ligand dissociation, the fluorescent signal remains sufficiently high to clearly distinguish the specific interaction from the background, ensuring reliable data acquisition even during extended plate processing. Taken together, these results demonstrate the construction of a specific assay platform for studying the PD‐1/PD‐L1 interaction.

### 3.2. Quantification of PD‐1/PD‐L1 Blockade Using an Anti‐PD‐L1 mAb

To evaluate the capacity of our assay to detect the blockade of the PD‐1/PD‐L1 protein–protein interaction, we tested a monoclonal anti‐PD‐L1 antibody (αPD‐L1 mAb) that was previously shown to inhibit this interaction [3], alongside an isotype IgG control as positive and negative controls, respectively. In the presence of the αPD‐L1 antibody, the gMFI was reduced; the same was not observed with an isotype control (Figure 2A,B). The general gating strategy used for the bead‐based assays is shown in Figure S1. To evaluate the dose inhibition, αPD‐L1 mAb and IgG isotype were titrated from 0.1 to 15 µg/mL. αPD‐L1 mAb induced a concentration dependent inhibition of PD‐L1–AF488 binding, as measured by both positive beads (Figure 2E) and the gMFI (Figure 2F). No inhibition was observed with the isotype control across any concentration tested (Figure 2C,D). Based on these results, the concentration of 12.5 µg/mL of PD‐L1 AF488 was selected for all subsequent experiments, as it provided a robust signal window for detecting competitive blockade.

Figure 2Blockade of the PD‐1/PD‐L1 interaction by an αPD‐L1 monoclonal antibody (mAb). (A) Schematic representation of the blocking assay. The αPD‐L1 antibody binds to the fluorescent PD‐L1 protein, preventing its binding to the PD‐1 protein conjugated on the bead surface. (B) Representative flow cytometry histogram showing the reduction in geometric mean fluorescence intensity (gMFI) of the PD‐L1 AF488 binding on PD‐1 beads in the presence of increasing concentrations of the αPD‐L1 antibody. The PD‐L1 AF488 concentration used was 12.5 µg/mL. (C, D) A nonblocking human IgG isotype control antibody was titrated using 0.1, 1, 5, 10, and 15 µg/mL against 0.75, 1.56, 3.12, 6.25, and 12.5 µg/mL concentrations of PD‐L1 AF488. The isotype control showed no inhibitory effect on the PD‐1/PD‐L1 interaction, as evaluated by (C) percentage of binding and (D) geometric mean fluorescence intensity (gMFI). (E, F) Titration of the blocking monoclonal αPD‐L1 antibody under the same conditions demonstrated dose‐dependent inhibition of the PD‐1/PD‐L1 interaction. The reduction in binding was measured by (E) percentage of binding and (F) gMFI. Representative data from three independent experiments with *n* = 3.

**Figure:**
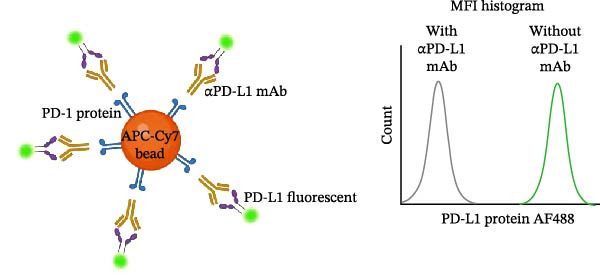
(A)

**Figure:**
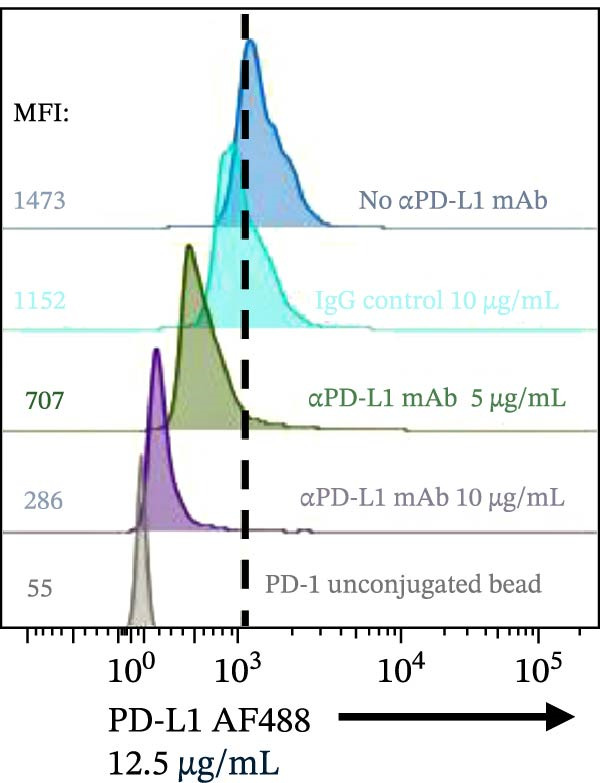
(B)

**Figure:**
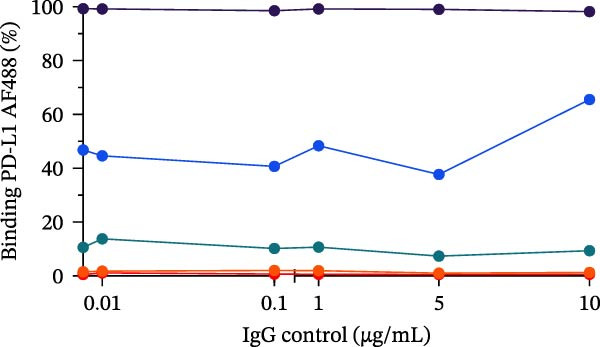
(C)

**Figure:**
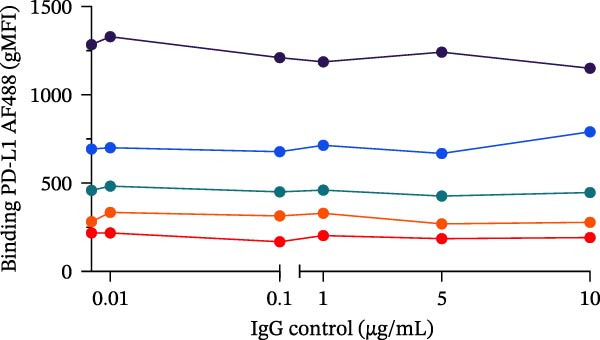
(D)

**Figure:**
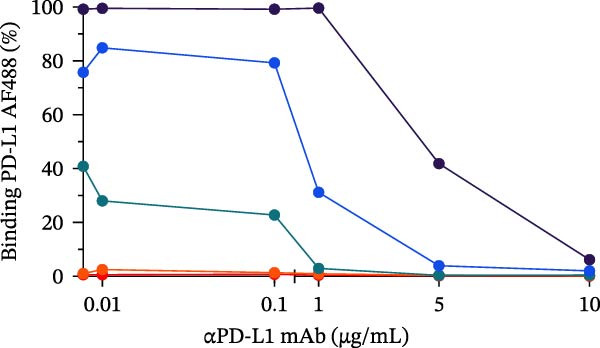
(E)

**Figure:**
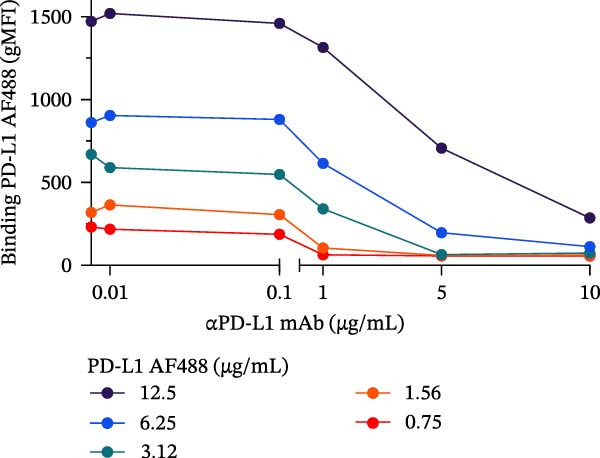
(F)

### 3.3. Assessment of Intra‐ and Inter‐Assay Precision

We next evaluated the analytical precision of the bead‐based PD‐1/PD‐L1 blockade assay by determining intra‐ and inter‐assay variability in a single run and across three different days, respectively. The results, summarized in Table 1 and Figure 3, demonstrate good overall repeatability. When analyzing the normalized binding signal (gMFI/gMFI_max_), the mean intra‐assay %RSD ranged from 4.68% to 22.45%. As expected, higher variability was observed at antibody concentrations corresponding to the lower signal plateau, where maximal blockade occurs. However, when the data were transformed to reflect the inhibition rate (1 ‐ gMFI/gMFI_max_), the %RSD values were noted to be artificially high at low antibody concentrations. Therefore, the most representative measure of the intra assay precision is observed in 1.0 −10.0 µg/mL αPD‐L1 range, which showed low variability between 0.6% and 6.0% (Table 1).

**Table 1:** Intra‐assay and inter‐assay precision of the bead‐based assay.

αPD‐L1 mAb (µg/mL)	Mean binding (gMFI/gMFI_max_) (*n* = 6)	Intra‐assay mean %RSD (*n* = 6)	Inter‐assay %RSD (*n* = 3)	Mean inhibition (1 − gMFI/gMFI_max_) (*n* = 6)	Intra‐assay mean %RSD (*n* = 6)	Inter‐assay %RSD (*n* = 3)
0.1	0628	4681	3202	0372	7897	59.865
1	0540	5298	6690	0461	6207	19.365
5	0191	9139	25,310	0809	2155	9.520
10	0025	22,450	69,648	0975	0568	2.369
15	0000	0000	73,538	1000	0000	2.049

*Note*: Precision, measured as percent relative standard deviation (%RSD), is shown for binding and inhibition metrics based on intra‐assay (*n* = 6) and inter‐assay (*n* = 3) data.

Figure 3Evaluation of intra and inter‐assay precision of the PD‐L1 blockade assay. (A) PD‐L1 binding inhibition curves from six replicates within a single run. (B) Comparison of inhibition curves from three independent runs. IC_50_ values were calculated for each run using nonlinear regression analysis. Data represent mean ± SD. Representative data from three independent experiments with *n* = 3.

**Figure:**
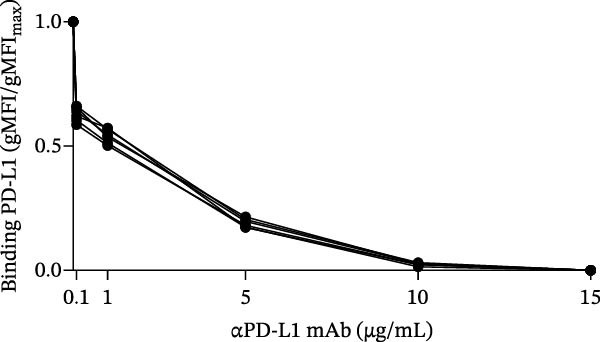
(A)

**Figure:**
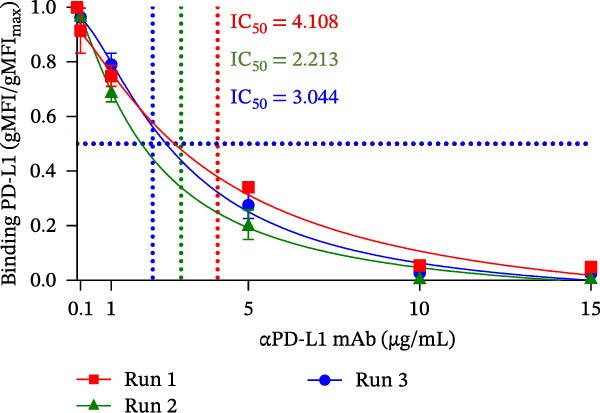
(B)

Inter‐assay reproducibility showed a consistent trend dependent on the chosen normalization method. As expected, the inter‐assay %RSD calculated from the binding signal (gMFI/gMFI_max_) increased as the signal approached zero. In contrast, when calculated from the inhibition rate (1 ‐ gMFI/gMFI_max_), the %RSD was highest at low inhibition and progressively decreased as the blockade effect increased (Table 1, Figure 3B). The half‐maximal inhibitory concentration (IC_50_) values calculated from each run were 4.108 µg/mL, 2.213 µg/mL, and 3.044 µg/mL, respectively, yielding a mean IC_50_ of 3.122 µg/mL and an inter‐assay %RSD of 30.4%. Taken together, these results demonstrate that the bead‐based assay is both precise and reproducible, making it a suitable tool for quantitative characterization of blocking antibodies.

### 3.4. Functional Validation of PD‐L1 Blockade in a T Cell Activation Assay

To determine whether the blockade of PD‐1/PD‐L1 detected by the bead‐based assay translates into a functional cellular response, we established a PD‐L1‐dependent T cell suppression model using a coculture system (Figure 4 and Figure S4). The complete gating strategy used to analyze this assay is detailed in Figure S3. First, a stable cell line overexpressing human PD‐L1 was generated by transfecting Expi293F cells, with around 80% of PD‐L1 expression confirmed by flow cytometry (Figure 4A,B). When cocultured with primary T cells (Figure 4C), PD‐L1^+^ Expi cells significantly suppressed T cell activation (Figure 4D). Stimulation of T cells with anti‐CD3 and anti‐CD28 antibodies induced robust upregulation of the activation marker CD69 and the exhaustion marker PD‐1 (Figure 4D). Moreover, when activated T cells were cocultured with Expi PD‐L1^+^ cells, the CD69 was significantly downregulated on T cells (*p*  < 0.0001) (Figure 4D), as well as the IFN‐γ production (*p*  < 0.0001) and proliferation capacity (*p* = 0.0014) (Figure S4A).With this system validated, the functional consequence of PD‐L1 blockade was tested. The αPD‐L1 mAb was added to the coculture at a concentration of 3 µg/mL, which approximates the mean IC_50_ (3.122 µg/mL) determined previously by the bead‐based assay. Treatment with αPD‐L1 mAb was able to reverse the PD‐L1‐mediated CD69 suppression, leading to a significant restoration of CD69 expression to around 23% (*p* = 0.0001) when compared to the IgG control (Figure 4E). In addition, a tendency to increase, with no significant effect, was observed for IFN‐γ production (*p* = 0.2907) and proliferation via Ki‐67 staining (*p* = 0.2131) (Figure S4B). We could also see the same profile of activation upon αPD‐L1 treatment in both CD4^+^ and CD8^+^ T cells (Figure S5). The CD69 recuperation confirms that the blocking activity identified in the bead‐based assay translates directly into an effect in an early functional cellular state, thus validating the results of our initial screening tool.

Figure 4Functional validation of the αPD‐L1 antibody in a T cell coculture assay. (A) Schematic representation of the generation of Expi293F cells overexpressing the human PD‐L1 protein (PD‐L1^+^ cells). (B) Flow cytometry analysis confirming high PD‐L1 expression (~82%) on transfected cells compared to the nontransfected control. (C) Illustrative diagram of the functional assay, where activated T cells are suppressed by PD‐L1^+^ cells and treated with the αPD‐L1 antibody or an IgG control. (D) Validation of the immunosuppressive effect in coculture. Activated T cells were cultured either alone (control) or in the presence of PD‐L1^+^ cells for 18 h. The presence of PD‐L1^+^ cells significantly reduced the expression of the early activation marker CD69 (left panel, *p*  < 0.0001) and increased PD‐1 expression (right panel, *p*  < 0.0001) on T cells compared to the T cell‐only condition. (E) Treatment with the αPD‐L1 antibody at a concentration of 3 µg/mL significantly restore the CD69 levels on T cells (*p* = 0.0001) compared to the IgG control. Bar graphs represent the mean ± standard deviation of replicates. *p*‐Values were calculated by one‐way ordinary ANOVA (D) and *t*‐test (E). Data represent mean ± SD. Representative data from three independent experiments with *n* = 3.

**Figure:**
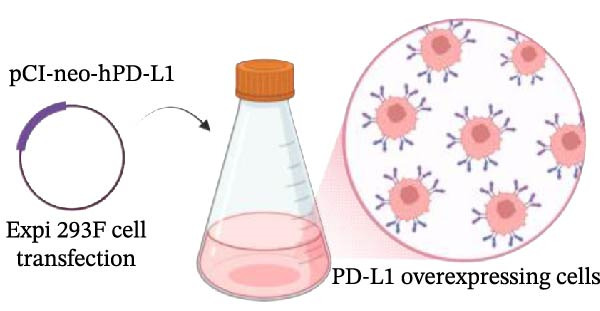
(A)

**Figure:**
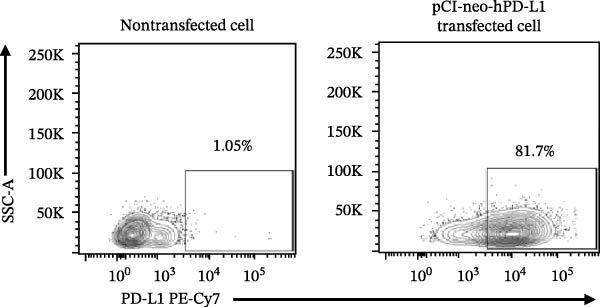
(B)

**Figure:**
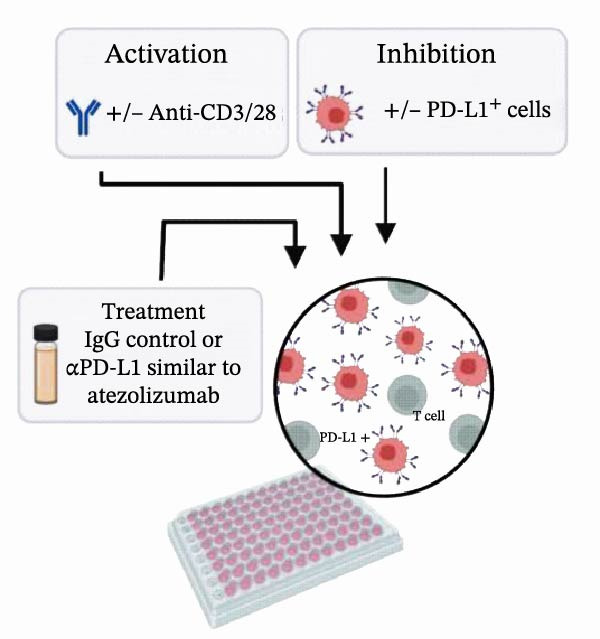
(C)

**Figure:**
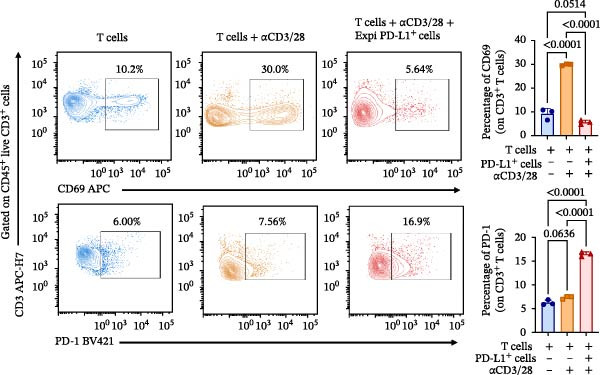
(D)

**Figure:**
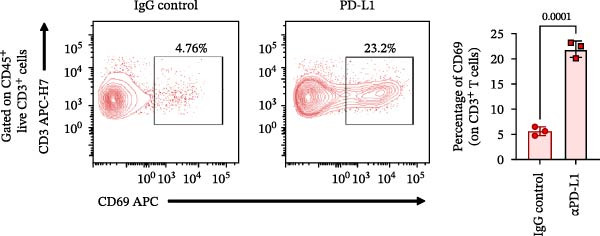
(E)

### 3.5. Validation With Additional Controls

To confirm the broad utility and specificity of the platform, we extended our analysis to include avelumab, another clinically relevant anti‐PD‐L1 antibody (Figure 5A). Both atezolizumab and avelumab exhibited dose‐dependent inhibition profiles (Figure 5B). Figure 5C exemplifies the capability of the assay in detecting the blockade at 5 µg/mL when compared to a nonblocking anti‐PD‐L1 mAb or an IgG control, atezolizumab (*p* = 0.0081) and avelumab (*p*  < 0.0001) significantly reduce the signal. Notably, at 5 µg/mL, the assay was able to significantly differentiate between blocking potencies of the therapeutic antibodies (*p* = 0.0242) (Figure 5D), highlighting its sensitivity to distinct affinities.

Figure 5Specificity and validation with therapeutic anti‐PD‐L1 antibodies. (A) Schematic representation of blocking efficiency comparison between different therapeutic anti‐PD‐L1 antibodies. (B) PD‐1‐conjugated beads were incubated with increasing concentrations of atezolizumab, avelumab, anti‐PD‐L1 nonblocking mAb, or IgG control in the presence of fluorescent PD‐L1. (C) Comparison of percentage of blocking (%) at 5 µg/mL showing that while atezolizumab and avelumab inhibit the interaction, the nonblocking anti‐PD‐L1 antibody does not, maintaining a signal similar to the IgG control. (D) Comparison of blockade capacity between atezolizumab and avelumab at 5 µg/mL. Data represent mean ± SD. Representative data from two independent experiments with *n* = 2.

**Figure:**
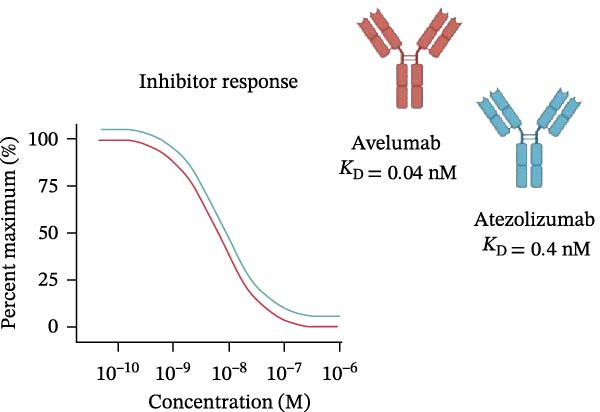
(A)

**Figure:**
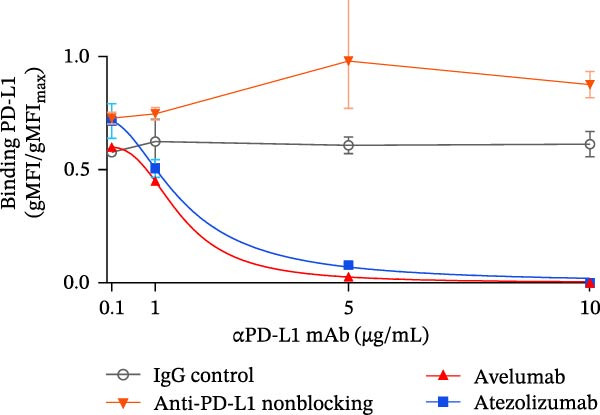
(B)

**Figure:**
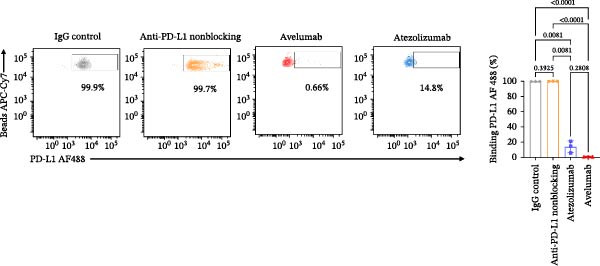
(C)

**Figure:**
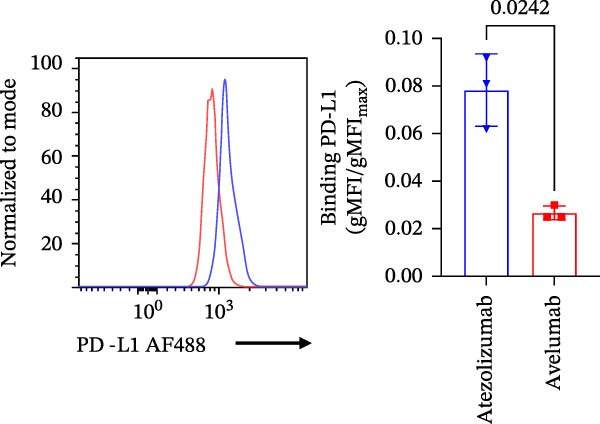
(D)

## 4. Discussion

In this study, we present a bead‐based flow cytometry assay for quantifying the blockade of the PD‐1/PD‐L1 immune checkpoint, building upon our previous work developing surrogate neutralization platforms for viral targets, including SARS‐CoV‐2 [9]. While utilizing a similar configuration, the application of this platform to the PD‐1/PD‐L1 axis offers distinct novelty; unlike the high‐affinity binding in viral neutralization, this approach addresses the specific kinetic challenges of immune checkpoint interactions. The implementation of analytical tools that are scalable, reproducible, and mechanistically informative remains critical for accelerating the development of immunotherapeutics, particularly checkpoint inhibitors [10, 11]. While functional cell‐based assays are essential for confirming biological activity, they are often complex and time‐consuming[12]. Our assay addresses this gap by providing a rapid, sensitive, and cost‐effective platform suitable for the initial screening and characterization of candidate mAbs. Our approach of using polystyrene beads coupled with a recombinant receptor to measure binding inhibition by flow cytometry aligns with methodologies that have been applied in other fields, such as viral serology and diagnostics [13–15]. For instance, [14] and Lopez et.al.[15], used a similar multiplexed bead array to evaluate the neutralizing capacity of antibodies against SARS‐CoV‐2 variants, highlighting the multiplexing capacity of this platform. The principle of measuring the blockade of a protein–protein interaction is a cornerstone of surrogate neutralization assays, which have been reviewed as valuable alternatives to live virus or cell‐based tests[16]. Our work extends this principle to the field of immune checkpoints inhibition. A study by [17] also developed a bead‐based assay for ICIs, specifically targeting PD‐1 and TIGIT, but using magnetic beads. A key finding of their work was that the sensitivity of the assay for the PD‐1/PD‐L1 axis was significantly higher when the ligand (PD‐L1) was immobilized on the bead and PD‐1 was used as the analyte (soluble ligand/bead immobilized receptor—sR/bL) assay format [17]. Our current assay immobilizes the receptor PD‐1 on the bead (sL/bR format). An important distinction, however, is that their validation used an anti‐PD‐1 antibody, while our study used an anti‐PD‐L1 antibody. This is significant because the choice of orientation determines whether the antibody targets a soluble or an immobilized protein. In the most sensitive format for Meng et al. [17] (sR/bL), their anti‐PD‐1 antibody targeted the soluble PD‐1 protein. Interestingly, in our sL/bR format, our anti‐PD‐L1 antibody also targets the soluble component (the fluorescent PD‐L1). This may suggest that assay sensitivity could be improved when the antibody can access its target in solution, potentially avoiding issues of steric hindrance or conformational changes on the bead surface. Therefore, exploring the reverse orientation in our system could be a valuable approach to evaluating if immobilizing PD‐L1 would further enhance the detection of our specific anti‐PD‐L1 antibody. This is one of our study limitations, and more experiments are necessary for these further confirmations. When comparing the signal report method, notably, the gMFI readout appeared to be more sensitive than the percentage of binding. An inhibition concentration dependence was observed via gMFI across all tested concentrations of the PD‐L1 AF488. However, the percentage of the binding metric only revealed this inhibitory pattern from the concentration 3.12 µg/mL (Figure 2C–F). Beyond establishing sensitivity metrics, a critical requirement for assay utility is the ability to discriminate between antibodies with varying blocking potencies. The addition of data comparing atezolizumab (*K*_D_ around 0.4 nM [18]) and avelumab (*K*_D_ around 0.04 nM [19]) (Figure 5) adds this layer of utility. The platform resolved the inhibitory curves of these high‐affinity binders, demonstrating its sensitivity to quantify relative potencies. Importantly, the assay clearly differentiated these potent inhibitors from a nonblocking anti‐PD‐L1 antibody, which, despite binding to the target, failed to disrupt the PD‐1/PD‐L1 interaction. This confirms that the system can effectively stratify candidates based on their functional mechanism of action, distinguishing functional neutralization from surface binding. A strength of our study is the validation of our findings on a bead‐based assay with a relevant human T cell activation in vitro model. We demonstrated that the αPD‐L1 antibody, at a concentration approximating the mean IC_50_ derived from our bead‐based assay (~3 µg/mL), restored the expression of the early activation marker CD69 on T cells. This is evidence that the blocking activity measured by our screening tool translates into a tangible functional outcome. This step is essential for bridging the gap between a simple binding assay and a complex biological system, a validation strategy also employed in the development of other surrogate assays where bead‐based results are correlated with cell‐based infection or neutralization [20, 21]. While our single point cellular validation at the IC_50_ serves as a proof‐of‐concept, we acknowledge its limitations. The lack of restoration of IFN‐γ and Ki‐67 is likely due to the early timepoint of analysis (18 h), as these are markers of a more sustained effector response. As a future perspective, generating a full dose response curve in the in vitro cellular model would be of significant value. This would enable a direct correlation analysis between the percentage of inhibition measured in the bead‐based assay and the percentage of CD69, as well as IFN‐γ and Ki‐67, restoration in the coculture assay across multiple concentrations and time points. In summary, we describe a precise, reproducible bead‐based flow cytometry assay for the characterization of PD‐1/PD‐L1 blocking antibodies. This platform is a rapid and reliable tool for antibody screening, bridging biophysical readouts with functional immune outcomes. Its integration into the immunotherapy development pipeline may help streamline candidate evaluation, inform lead selection, and support quality control during manufacturing and release testing.

## Author Contributions

**Veridiane M. Pscheidt**: writing – original draft, methodology, formal analysis, investigation, conceptualization. **Rodrigo B. Gassen**: methodology, supervision, writing – review and editing, investigation, validation, data curation, formal analysis. **José E. Sacconi Nunes**: methodology, supervision, validation. **Deise do Nascimento de Freitas**: methodology, supervision. **Valdir Barth Jr** **, Fernanda B. Frozza, and Milena D. Santos**: methodology. **Claudia P. Nunes**: methodology, resources. **Cristina B. C. Bonorino**: project administration, conceptualization, writing – review and editing, supervision.

## Funding

This study was funded by the Brazilian Ministry of Health (PRONON/DECIT, Grant 25000.172780/2019‐38) and the Foundation for Research Support of the State of Rio Grande do Sul (FAPERGS/RITES, Grant 22/2551‐0000388‐5).

## Conflicts of Interest

The authors declare no conflicts of interest.

## Supporting Information

Additional supporting information can be found online in the Supporting Information section.

## Supporting information


**Supporting Information** Figure S1. Flow cytometry gating strategy for the bead‐based binding assay. The main bead population was first identified using its characteristic fluorescence in the APC‐Cy7 channel. Subsequently, the binding of PD‐L1 AF488 was quantified on the gated bead population. Figure S2. Gating strategy for CD3^+^ cell sorting and confirmation of postsort population purity. Figure S3. Gating strategy for the identification of viable, activated (CD45^+^CD3^+^ and CD69^+^or IFN‐γ^+^ or Ki‐67^+^) T cells. Figure S4. Analysis of T cell effector markers expression following αPD‐L1 treatment. (A) Validation of the suppression system, showing that coculture with PD‐L1^+^ cells significantly reduces the expression of the IFN‐γ and Ki‐67 on T cells (*p*  < 0.0001 and *p* = 0.0014). (B) Treatment with the αPD‐L1 antibody at a concentration of 3 µg/mL did not significantly restore the IFN‐γ and Ki‐67 levels on T cells (*p* = 0.2907 and *p* = 0.2131) compared to the IgG control. Bar graphs represent the mean ± standard deviation of replicates. *p*‐Values were calculated by ordinary ANOVA (A) and *t*‐test (B). Data represent mean ± SD. Representative data from three independent experiments with *n* = 3. Figure S5. T cells cocultured with PD‐L1^+^ cells were treated with 3 µg/mL of anti‐PD‐L1 antibody or an IgG isotype control. The expression of the early activation marker CD69 was analyzed within the CD4^+^ and CD8^+^ T cell subsets. Treatment with anti‐PD‐L1 significantly increased the frequency of CD69^+^ cells in both subpopulations compared to the IgG control. Data represent mean ± SD. Statistical significance was determined by Student’s *t*‐test (*p*  < 0.05). Representative data from three independent experiments with *n* = 3. Table S1. Antibody panel for T cell coculture assay.

## Data Availability

The data that support the findings of this study are available from the corresponding author upon reasonable request.
