# Supplementary material for: A Bead‐Based Screening Platform for Identifying Monoclonal Antibodies That Disrupt PD‐1/PD‐L1 Interactions
Source: J Immunol Res. 2026 Mar 31;2026:5189659. doi: 10.1155/jimr/5189659 (PMC13140367; doi:10.1155/jimr/5189659)
Supplement: Supplementary file 1 — Supporting Information Figure S1. Flow cytometry gating strategy for the bead‐based binding assay. The main bead population was first identified using its characteristic fluorescence in the APC‐Cy7 channel. Subsequently, the binding of PD‐L1 AF488 was quantified on the gated bead population. Figure S2. Gating strategy for CD3+ cell sorting and confirmation of postsort population purity. Figure S3. Gating strategy for the identification of viable, activated (CD45+CD3+ and CD69+or IFN‐γ+ or Ki‐67+) T cells. Figure S4. Analysis of T cell effector markers expression following αPD‐L1 treatment. (A) Validation of the suppression system, showing that coculture with PD‐L1+ cells significantly reduces the expression of the IFN‐γ and Ki‐67 on T cells (p < 0.0001 and p = 0.0014). (B) Treatment with the αPD‐L1 antibody at a concentration of 3 µg/mL did not significantly restore the IFN‐γ and Ki‐67 levels on T cells (p = 0.2907 and p = 0.2131) compared to the IgG control. Bar graphs represent the mean ± standard deviation of replicates. p‐Values were calculated by ordinary ANOVA (A) and t‐test (B). Data represent mean ± SD. Representative data from three independent experiments with n = 3. Figure S5. T cells cocultured with PD‐L1+ cells were treated with 3 µg/mL of anti‐PD‐L1 antibody or an IgG isotype control. The expression of the early activation marker CD69 was analyzed within the CD4+ and CD8+ T cell subsets. Treatment with anti‐PD‐L1 significantly increased the frequency of CD69+ cells in both subpopulations compared to the IgG control. Data represent mean ± SD. Statistical significance was determined by Student’s t‐test (p < 0.05). Representative data from three independent experiments with n = 3. Table S1. Antibody panel for T cell coculture assay. [file JIMR-2026-5189659-s001.docx]

**Supplementary material for the manuscript:**

**“A bead-based screening platform for identifying monoclonal antibodies that disrupt PD-1/PD-L1 interactions”**


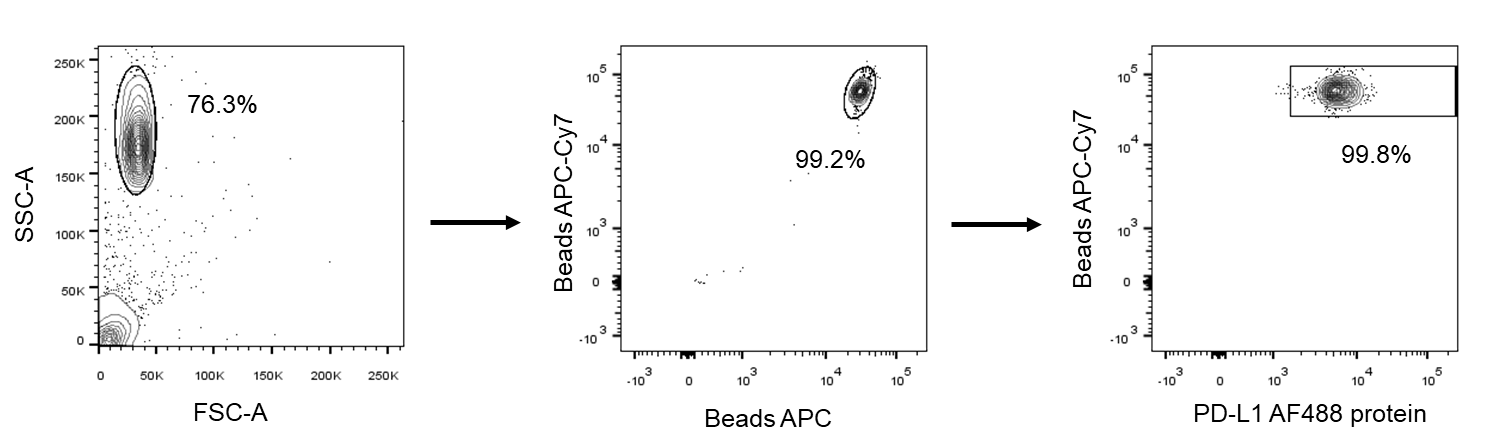


**Supplementary Figure 1.** Flow cytometry gating strategy for the bead-based binding assay. The main bead population was first identified using its characteristic fluorescence in the APC-Cy7 channel. Subsequently, the binding of PD-L1-AF488 was quantified on the gated bead population.


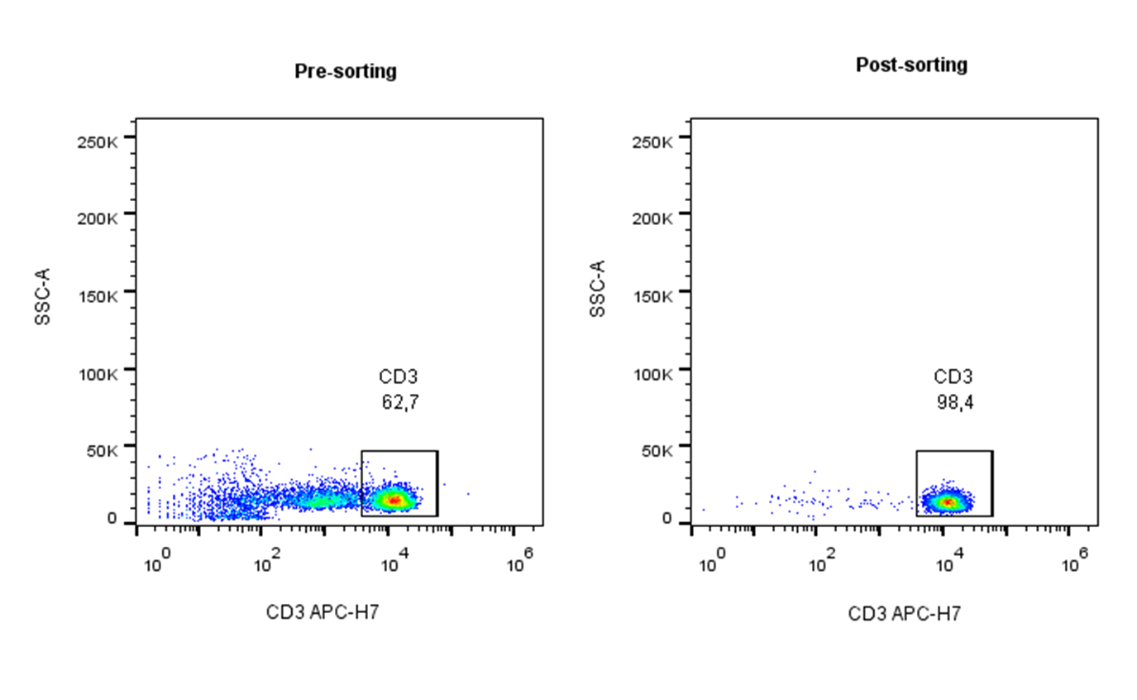


**Supplementary Figure 2.** Gating strategy for CD3⁺ cell sorting and confirmation of post-sort population purity.


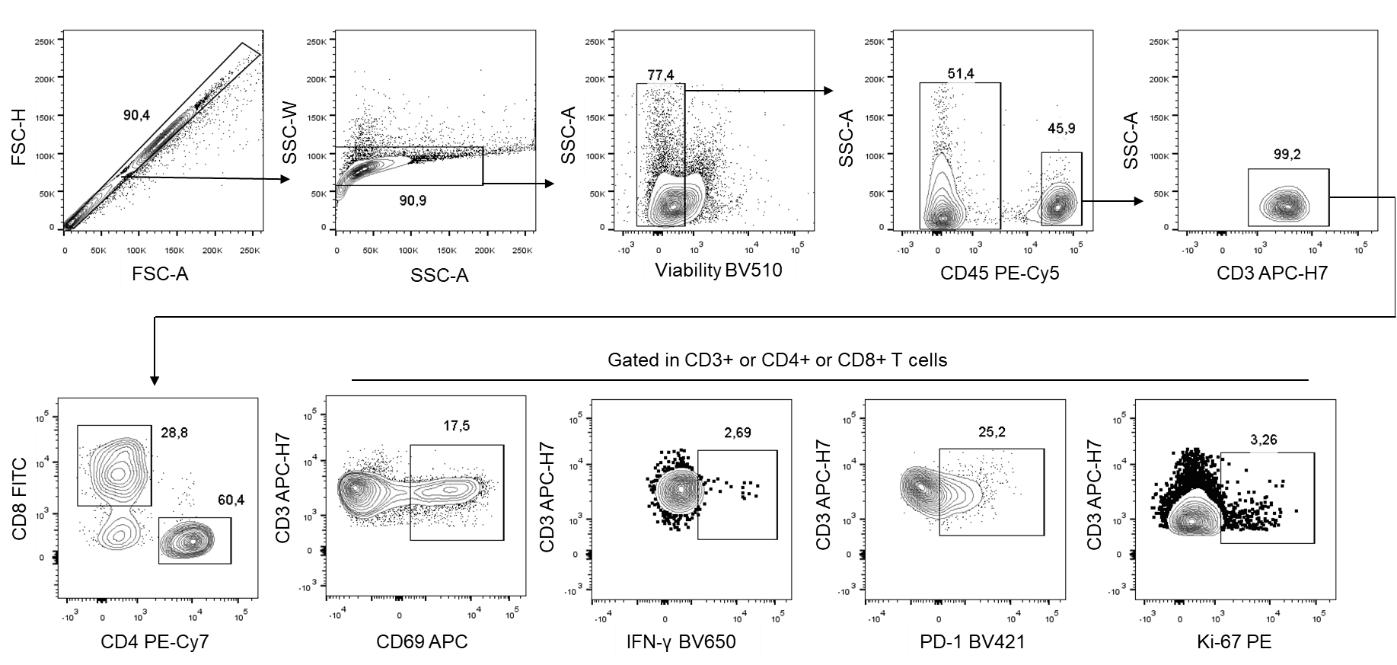


**Supplementary Figure 3.** Gating strategy for the identification of viable, activated (CD45⁺CD3⁺ and CD69⁺or IFN-γ⁺ or Ki-67⁺) T cells.


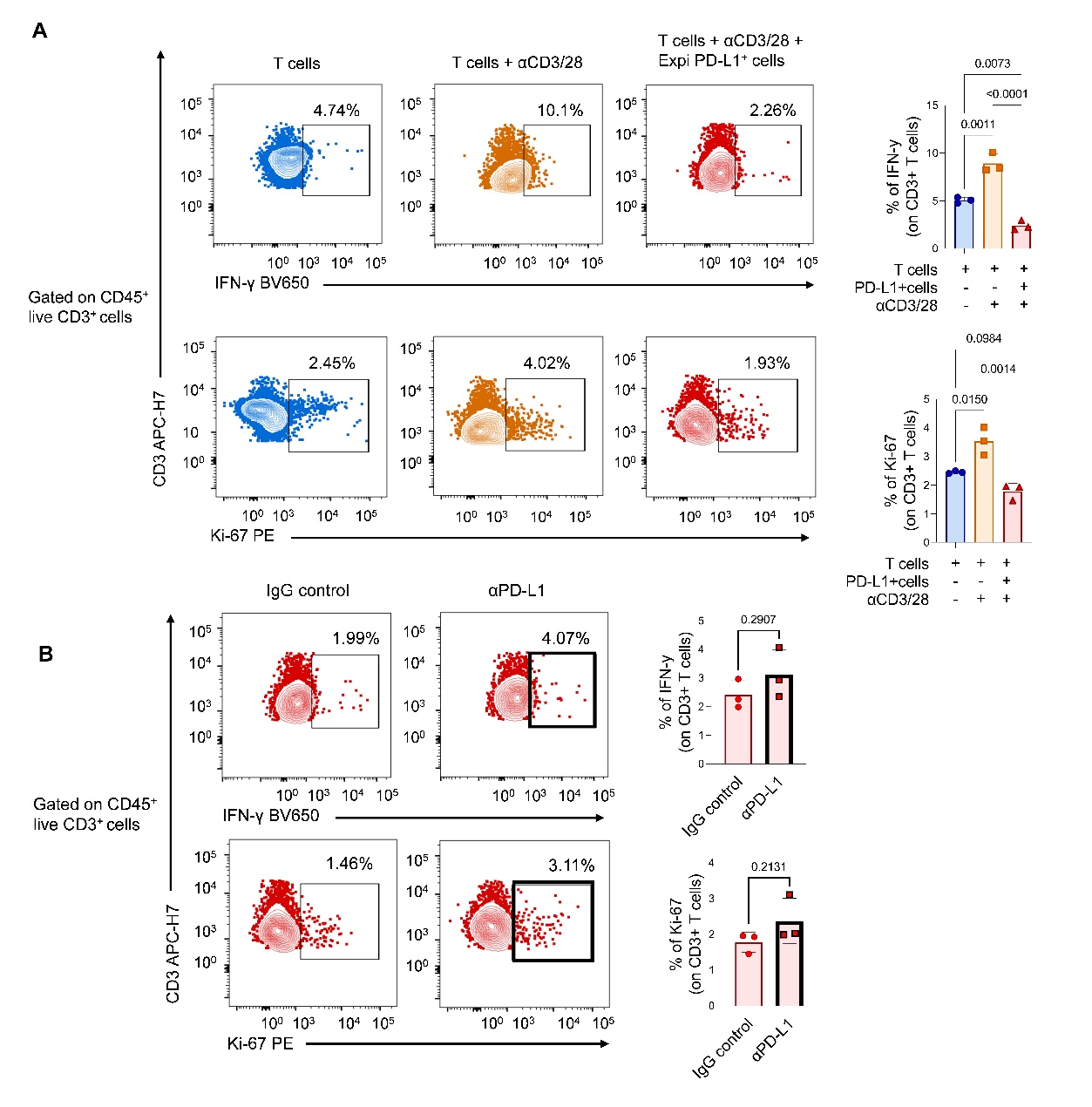


**Supplementary Figure 4.** Analysis of T cell effector markers expression following αPD-L1 treatment. (A) Validation of the suppression system, showing that co-culture with PD-L1+ cells significantly reduces the expression of the IFN-γ and Ki-67 on T cells (p<0.0001 and p=0.0014). (B) Treatment with the αPD-L1 antibody at a concentration of 3 µg/mL did not significantly restore the IFN-γ and Ki-67 levels on T cells (p=0.2907 and p=0.2131) compared to the IgG control. Bar graphs represent the mean ± standard deviation of replicates. P-values were calculated by ordinary ANOVA (A) and t-test (B).


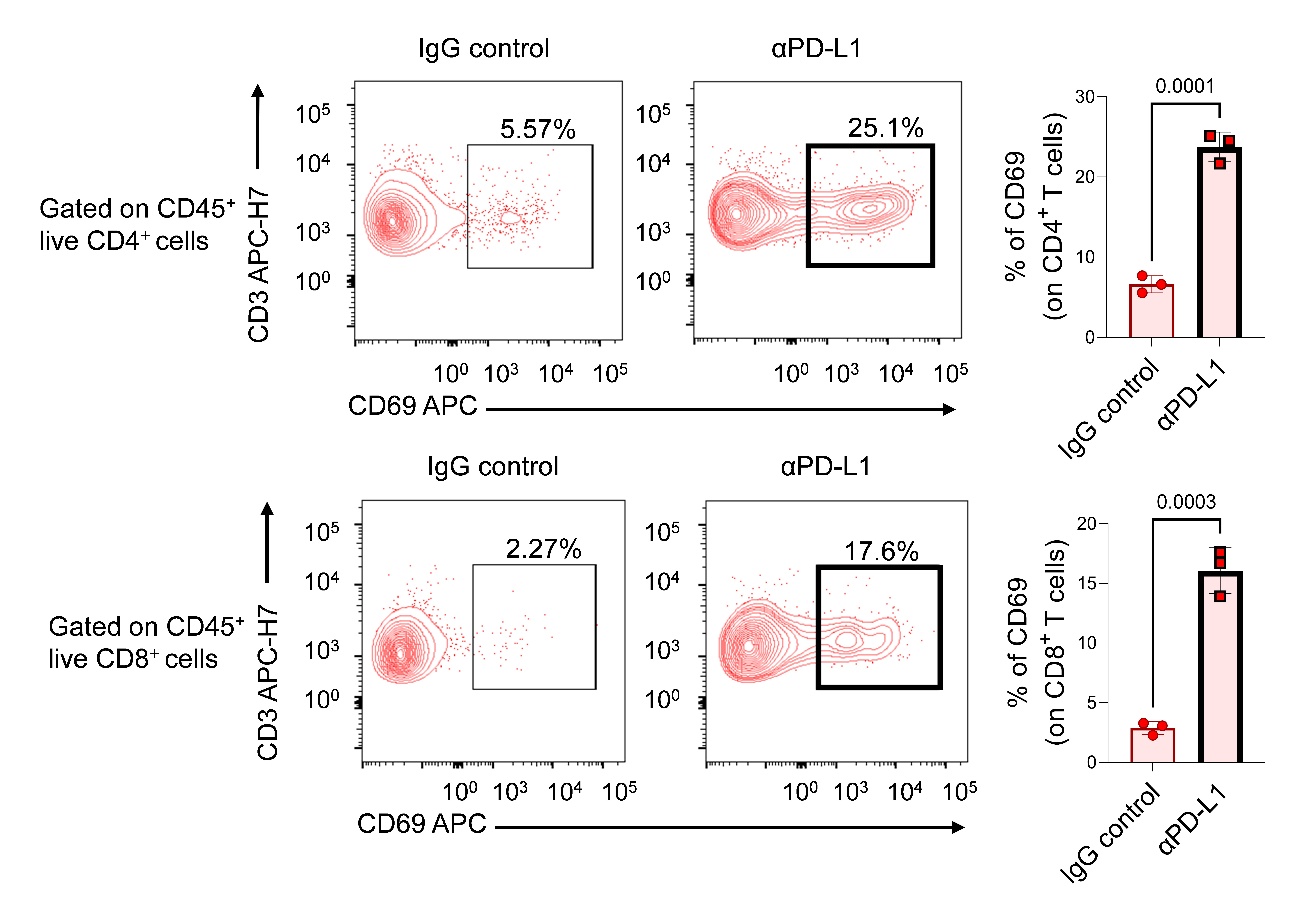


Supplementary Figure 5. T cells co-cultured with PD-L1+ cells were treated with 3 µg/mL of anti-PD-L1 antibody or an IgG isotype control. The expression of the early activation marker CD69 was analyzed within the CD4+ and CD8+ T cell subsets. Treatment with anti-PD-L1 significantly increased the frequency of CD69+ cells in both subpopulations compared to the IgG control. Data represent mean ± SD. Statistical significance was determined by Student’s t-test (p < 0.05). Representative data from three independent experiments with n = 3.

**Supplementary Table 1.** Antibody panel for T cell co-culture assay.

| Target | Fluorochrome | Clone | Volume/well (μL) | Brand/Cat. No. |
| --- | --- | --- | --- | --- |
| CD45 | PE-Cy™5 | HI30 | 1 | BD/555484 |
| CD3 | APC-H7 | SK7 | 2 | BD/641415 |
| CD4 | PE-Cy™7 | SK3 | 1 | BD/557852 |
| CD8 | FITC | HIT8a | 1 | BD/555634 |
| CD69 | APC | FN50 | 2 | BD/555533 |
| CD279 | BV421 | MIH4 | 2 | BD/564323 |
| IFN-γ | BV650 | 4S.B3 | 3 | BD/563416 |
| Ki-67 | PE | B56 | 2 | BD/567719 |
| Viability Dye | FV510 | - | 150 (1:1000) | BD/564406 |
